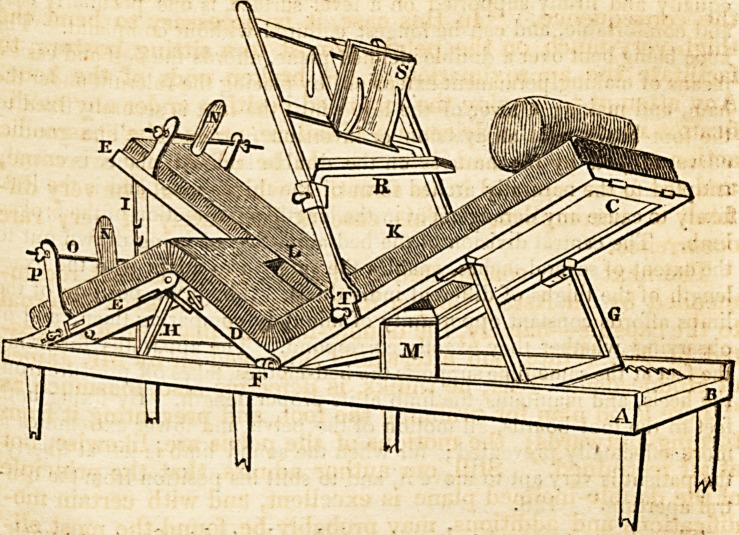# Mr. Earle's Practical Observations in Surgery

**Published:** 1824-06-01

**Authors:** 


*Practical Observations in Surgery.* By HENRY EARLE, F.L.S. Assistant Surgeon to St. Bartholomew's Hospital, and Surgeon to the Foundling. Octavo, pp. 230. Underwoods, 1823.

IN our Number (15) for December last, we were only able to take up the litigated question of "*union or non-union*" of the neck of the femur within the capsular ligament. We were then obliged to pass over the other subjects treated of in Mr. Earle's volume. We now return to the work in question, in order to make our readers more particularly acquainted with its contents.

At page 102 of Mr. Earle's work, this ingenious surgeon gives up discussion, and proceeds to the treatment of fractures at the upper part of the thigh-bone. He remarks, that the treatment of fracture, whether within or external to the capsule, is the same as in any other fracture—namely, to reduce the limb to its proper length, to adapt the broken surfaces together, and to maintain them so. This rule is easy in theory, but difficult in practice. Thus, where the neck of the femur is broken, no lateral pressure can have any effect in restraining the motion of the broken bones; nor is it possible to place any splint sufficiently high on the inside of the limb to have any direct control over the fracture. Another difficulty arises from the head of the bone being so connected with the pelvis as to partake of every motion of the trunk—the consequence of which must be the continual disturbance of the broken bones.

"In treating these cases, it is essential to consider them as if the fracture existed between the pelvis and thigh; and so to connect the two that they become like one body, and move together. The other indications are to keep up moderate permanent extension, and to take care that the limb is in every respect in its proper relative situation, not only as to length but direction." 103.

Mr. Earle makes some pertinent remarks on the modes of treatment in most common use at the present day, beginning with that of Pott. We agree with our ingenious author, that the idea of relaxing *all* the muscles of a limb, by position, is perfectly visionary. The most powerful muscles, however, may be relaxed—or rather the longest, and, consequently, those most likely to exert a deleterious influence over the fracture, and most disposed to spasmodic contraction.

"I must confess that it has ever appeared to me that too much stress has been laid upon this action of muscles; and I cannot but think that, if they were the sole agents in displacing a fracture, it would be very immaterial in what position a limb was placed. To me it appears that the displacement of a fractured thigh depends, in the first instance, on the direction and continued operation of the force which causes the breach of continuity; or on the superincumbent weight of the trunk and pelvis; or on the injudicious mode in which a patient is removed after the receipt of an accident; or on some subsequent exertion, in attempting to use the limb or alter the position from that in which he fell. The muscles are but secondary agents: the broken spicula of bone, by lacerating them, render them irritable; they act spasmodically, and are no longer under the control of the will. When thus excited, they forcibly retain the bone in its wrong situation, and by their action considerably add to the deformity." 106.

This action of the muscles, Mr. Earle observes, is easily overcome by soothing the agitation into which they are thrown, by gently extending the limb, and restoring the broken bone as nearly as possible to its proper situation. By these means, the muscles will lose the disposition to retract the limb in 24 or 48 hours.

Mr. Earle avers, that he has never seen a case treated in the side position of Pott unattended with considerable lameness and eversion of the foot.

"The objections to this plan are, I think, manifold. In the first place the great difficulty, I will add almost impossibility, on a common hospital bed, of retaining a patient permanently on the side, is a very serious objection; and the slightest deviation from it causes the pelvis to gravitate, and turn more or less supine; carrying with it the upper portion of bone: the effect of this must be a permanent eversion of the leg and foot. But I will suppose that great care has been taken in preparing the bed, and every precaution used to keep the patient with the pelvis in the position above described; still, in fractures of the neck, or at the root of the trochanter, the whole weight of the pelvis resting upon the broken part, and bruised integuments, causes the most insufferable pain, and in old emaciated persons would soon produce gangrene; and should the broken surfaces not exactly correspond, all these evils would be increased tenfold." 108.

To these objections, Mr. Earle urges in addition, the irksomeness of the position, which cannot be varied—the loss of the use of the arm on the affected side—the want of any power to counteract the retraction of the limb—and the great difficulty of comparing the limbs together, to ascertain their lengths.

The straight or extended position, in fractures of the thigh-bone, is that which is usually employed on the Continent—and by several practitioners in this country. The object of all machines or apparatus for effecting this, is the same:—"to connect the thigh firmly with the pelvis, and keep up permanent extension of the broken limb." Mr. Earle has tried, or seen tried, all these contrivances, and thinks them liable to many and serious objections.

"In the first place, the perfectly supine position which is required to restrain the tendency of the body and pelvis to sink to the lower part of the bed, is irksome and unfavourable to the taking food, and still more so to the expulsion of the fæces and urine. In several cases I have known the employment of the catheter necessary both in the male and female; and very painful retention of urine, with all its distressing attendants, has been the consequence of neglect.

"In removing the fæces, more or less motion must be given to the pelvis; and as the other limb is left at liberty, there will, in all cases, be more or less power of moving the pelvis as the bandages become loosened; and consequently the upper portion of the fracture will be liable to be displaced, which cannot but be unfavourable to bony union.

"The prodigious number of bandages in Desault's apparatus is productive of great trouble and inconvenience. The pressure which, in all the modifications of the long splint, is made across the groin causes great œdema of the whole limb, in consequence of the obstruction which is afforded to the circulation in the inguinal veins and absorbents. The bandages at the upper and inner part of the thigh are so liable to become soiled and wetted, particularly in the female, that distressing excoriation and ulceration, succeeded by enlargement of the glands, are by no means unfrequent consequences. But all these objections, by great care and assiduity on the part of the surgeon, may be counteracted, and in some measure obviated. There are, however, other objections, which have not hitherto met with the attention they merit, but which, I am confident, have, in many instances, contributed to retard the recovery, and produce permanent deformity, not only in fractures of the neck, but likewise of the shaft of the bone.

"In the application of the long splint, the two principal points of bearing are at the foot, and at the tuberosity and ramus of the ischium. Between these two parts the limb may be extended; and, by comparison with the other limb, the length of the two may appear exactly equal, and the surgeon may flatter himself that the broken bones are in perfect coaptation; but there is another very important circumstance to attend to, namely, the direction of the bone. This has been already pointed out in the anatomical description, where the great obliquity of the shaft of the bone, particularly in the female, has been dwelt upon.

"In Desault's apparatus, and, indeed, in all the applications of the long splint, the limb is bound to a perfectly straight body, which is placed at the outside of the thigh, and considerable pressure is made on the inner side of the knee; which, in fractures of the shaft of the bone, tends to destroy the natural obliquity of that part, and bring it more into a straight line, which is not only destructive to the symmetry of the limbs, but impedes progression, and renders the erect posture less secure.

"In fractures of the neck of the bone, pressure applied in the same direction will separate the lower edges of the fracture, and thus materially increase the difficulty of union. The forcible pressure from within outwards, which is made at the point of extension at the upper and inner part of the thigh, will often separate the broken portions of bone. The straight position is also objectionable, with reference to the arched form of the thigh; for I conceive it must be self-evident, if we take an arched and oblique body, in which there exists any breach of continuity, and attempt to forcibly confine it to a straight unyielding plane, that more or less displacement in the proper relative situation of the different parts must be the consequence. It is true, that, by the employment of graduated pads, the natural direction of the thigh-bone may be preserved; but this precaution is nowhere inculcated, and I have never seen it attended to." 113.

Of all the instruments which our author has seen, he thinks the one proposed by Mr. Hagedorn the least liable to objection. It is described in Cooper's First Lines of Surgery, p. 430. If there was superadded any simple contrivance for removing the fæces, without, in any degree, moving the pelvis, Mr. Earle would consider Hagedorn's apparatus as perfectly adapted to fulfil all the ends proposed. In this respect, however, it is defective.

There is one case of fracture, namely, just below the trochanter minor, where the psoas magnus and iliacus internus muscles often draw up, so forcibly, the upper portion towards the groin, as to cause a very evident projection at that part. If the bone should unite in that position, lameness and deformity would be the consequence. "In this case, it is necessary to bend the thigh very much on the pelvis, almost to a sitting posture, to facilitate the approximation of the broken ends of the bone. Any attempt to employ the extended position under any modification would be nearly certain of failure, unless the spasmodic action of the psoas and iliacus could be effectually overcome, and the bone replaced in its situation, which would be very difficult to accomplish." This case is fortunately of very rare occurrence.

The next plan commented on by Mr. Earle, is that recommended by Sir Astley Cooper in fractures of the neck external to the capsule—namely, the double-inclined plane, first suggested by Mr. White, and afterwards improved on by Mr. James of Hoddesden. This, he thinks, is defective also, inasmuch as "there is no plan for confining the foot, and preventing it from turning outwards; the motions of the pelvis are, likewise, not at all restrained." Still, our author admits, that the principle of the double-inclined plane is excellent, and with certain modifications and additions, may probably be found the most eligible mode of treating all fractures of the thigh.

It was in the year 1806, that a distressing case of fracture induced Mr. Earle to exert his ingenuity in contriving a double bed, an account of which was published by his father. The contrivance was honoured with a reward from the Society of Arts. By this contrivance, Mr. Earle gained one object which he considered of great importance, namely, a state of permanent rest for the whole trunk and extremities, while the additional comfort of cleanliness was afforded the patient. After describing several kinds of apparatus, which he tried with more or less success, Mr. Earle comes to the plan which he now offers to the profession as capable of fulfilling every desirable indication—being, at once, simple and easy of application—endurable for an indefinite length of time—fully adequate to maintain the pelvis quiet—extend the limb—and, lastly, cheap.

We have caused a wood-cut to be taken of one of Mr. Earle's beds, and present it on the next page for our readers' inspection.

"The apparatus* consists of a modification and improvement of the double inclined plane. The bed on which the patient is placed is divided into three portions, the upper one for the trunk, the short middle one for the thighs, and the lower division for the legs. These admit of being placed at various angles and in different positions, as will be best seen by a reference to the plate. The following are the advantages gained by this apparatus:—When the patient is placed on the bed, the pelvis will, from its own gravity, remain fixed at the bottom of the angle formed by the superior and central inclined planes; and the aperture made in the central part readily admits of the patient relieving himself, and being properly cleansed, without the least movement of either trunk or extremities. Should it be desirable, in young persons, or under particular circumstances, to secure the pelvis more firmly, it may be easily accomplished by two broad straps, brought from the edge of the aperture, and passed obliquely round the upper and outer part of the thighs; which should pass once round the pelvis, and be attached to buckles at the outer side of the mattress. By this simple plan the possibility of motion of the pelvis is prevented, and firm compression may be applied over the trochanter: this will, however, very rarely be required, as, generally speaking, the weight of the pelvis is sufficient to keep it steady; and no other bandage is requisite than that which secures the feet to the foot-boards. The position of the patient, namely, on the back, on a gently-inclined plane, with the thighs and legs half bent, and the whole

* "This apparatus was rewarded by the Society of Arts, &c. with their large gold medal, and is described in vol. xxxix. of their Transactions."

equally and firmly supported on a level surface, is one peculiarly easy and comfortable, and can be longest endured without complaint. The knee being bent over a double inclined plane, affords the best and easiest means of making permanent extension, by placing the fulcrum under the ham, and making a lever of the leg, whilst the foot is securely fixed to the foot-board, and all eversion or inversion prevented. The gradual curve, formed by the mattress on the double inclined plane, is exactly adapted to the naturally arched form of the thigh-bone, and is the least likely to cause any derangement in the length and direction of the broken limb. The central division of the bed admitting of being drawn out to the extent of several inches, enables the surgeon to adapt it to the exact length of the thighs of different individuals. The juxta-position of the limbs affords constant opportunity of minutely comparing them, and of observing whether they exactly correspond. The apparatus for fixing the feet at the same time supports the bed-clothes, takes off pressure from the heels, and maintains the limb at its proper length. By fixing both feet to the foot-boards, all motion of the pelvis and lower extremities is more effectually prevented; for when the sound limb is left at liberty, the patient is very apt to move it, and to shift his position from the central aperture." 128.

The above apparatus is not merely adapted to fractures within the articulation, but to other injuries and diseases, in which a state of permanent rest is essential to recovery, as diseases of the spine and hip, compound fractures of the thigh and leg, &c. It will be seen by the wood-cut, that a swing table and reading desk are added for the patient's use and amusement. We cannot do justice to this apparatus without adding the following directions from the author.

" The mattress should be either of horse-hair, or well stuffed with the best wool, and should be nailed round its edge, at the upper divison of the frame. A blanket and sheet should be separately strained over the mattress, and carefully sewed all round its edges: this will prevent any subsequent wrinkling, and by sewing first the blanket, and then the sheet, it is obvious that the latter may, if necessary, be detached, without at all disturbing the former. The whole apparatus is made narrow, both to facilitate the operations of the surgeon and nurse, in dressing or cleansing the patient, and to prevent him from shifting from the central aperture. Half a blanket, and a single breadth of sheeting, will, in all cases, be sufficient; and in fitting them to the central aperture, it is better to make a cross cut from the four corners, thus X, than to remove any part.

" The loose edges should then be turned down, and sewed at the lower part of the opening. By this plan any hardness of the edges of the aperture will be avoided. In fractures of the thigh, the length of the healthy limb should be accurately taken, and the central division of the bed should be drawn out, so as to make a slight degree of extension at the ham, when the limb is placed over the double inclined plane. The foot-board should likewise be placed at the proper distance to meet the feet. The patient may now be placed upon the bed, and the fundament should be exactly opposed to the central opening. In fractures through the cervix the pelvic bandage may be employed, but, generally speaking, the weight of the pelvis at the bottom of the inclined planes is sufficient to keep it steady; and the patient soon finds himself so easy and comfortable that he is very unwilling to move. Both feet should be secured to the foot-boards, either by bandages, or a pair of short cloth boots, made to lace in front, quite down to the toes. This plan will be found more comfortable than any common bandage, and the boot can be secured to the foot-board by screws or straps. In cases where retraction has taken place, the powerful extension obtained by placing the fulcrum under the ham, and securing the feet, will very soon reduce the limb to its proper length, and maintain it steadily in that position. In the cases in which I have employed it, I have not found it necessary to use any splints, even in fractures of the shaft of the bone; but, if requisite, they may be added. In compound fractures of the thigh or leg, where there is a probability of profuse discharge, it will be better to add some oil silk, and a draw-sheet under the part affected. When the fracture occurs immediately below the trochanter minor, and there is much spasmodic contraction of the psoas and iliacus muscles, it will be right to raise the superior and middle divisions of the bed very considerably, so as to place the thigh nearly at right angles with the body: this will completely approximate the fractured surfaces, which is with great difficulty accomplished by any other mode of treatment. As the extension in fractures occurring in any part of the thigh-bone is effected by the pressure on the calf of the leg, and in the ham, it is particularly necessary to adapt the central division of the bed to the exact length of the limb: when necessary to make any trifling alteration in this degree of extension, this is best effected by placing wedges of wood beneath that part of the mattress which supports the calf of the leg. The mattress being left loose at this part, readily admits of this being done without disturbing the patient." 134.

### Fracture of the Olecranon.

This chapter is chiefly disputative; but we shall endeavour to gather from it the practical matter and opinions which it is designed to convey.

In the summer of 1820, Mr. Earle was called to a gentleman who had been thrown out of his gig four hours previously, and whose elbow was swollen and extensively ecchymosed. Pronation, supination, flexion, and extension, could be spontaneously performed. Leeches were applied. On the 3d day he went to his house of business—but, on the 6th morning, he found so much difficulty in the motions of the joint that Mr. Earle was again sent for, and found that the olecranon was fractured.

"On bending the fore-arm, a separation between the olecranon and the shaft of the ulna could be evidently traced. This separation, however, was only in consequence of the removal of the lower portion in the act of flexion; the upper portion remained exactly in its proper relative situation, and there was not the slightest disposition in the triceps to retract it." 146.

Mr. Earle placed the limb, with the fore-arm slightly bent to an angle of about 160 degrees, as nearly as possible in the position in which the arms remain in a state of repose. This allowed of the most perfect coaptation of the fractured surfaces, without any forcible compression of their posterior broken edges. Splints of thick wetted pasteboard were adapted to the whole limb. The case went on most favourably, and in six weeks he was enabled to use the arm freely. The motions of it were quite restored, and the bone firmly united without any perceptible interval. The lateral motion of the detached portion was restrained by compresses and cross straps of adhesive plaster.

The only peculiarity attending this case, and which has induced Mr. Earle to give it publicity, is the power of extending the elbow with force up to the sixth day after the accident. The tendinous expansion which covers the olecranon, had not till then, he thinks, been torn through, and, consequently, had been a sufficient bond of union to allow of extension of the arm, and to hold the fractured portions together. Mr. Earle observes, that the possibility of such a state of things as above existing should be borne in mind, otherwise a surgeon might be led into error, as it is invariably stated by surgical writers, that a loss of the power of spontaneously extending the fore-arm is the constant and immediate consequence of a fracture of the olecranon. Another diagnostic mark of this fracture, as laid down by authors, is the degree of retraction of the superior portion, by the action of the triceps extensor cubili. In addition to the case above described, Mr. Earle avers that he has met with several others in which there was no retraction of the detached portion. In support of this statement, he quotes the words of Mr. Sheldon, who was himself the subject of an accident of this kind. Mr. Earle thinks fracture of the olecranon by the mere action of the muscles, independent of external violence, must be a very rare occurrence, as he can find no clear account of such a case on record.

"Certainly, in the event of such an accident, the retraction of the detached portion of bone, by the continued operation of the triceps, might be expected to follow. But in the more common mode in which this accident occurs, namely, by the direct application of violence to the base of the olecranon, with the arm in a state of flexion, the force does not necessarily displace the broken portions directly, nor does it follow that any consecutive retraction should take place from the spasmodic action of the triceps. As no violence is done to that muscle, there is no reason for its being thrown into spasm: and, generally speaking, any breach of continuity in a bone is accompanied with such a consciousness of inability to move the limb, that it requires a considerable effort on the part of the patient to will the muscles to act, and often the power of doing so is not at all under his control." 159.

Our author does not, however, deny the occurrence of such retraction, in some instances—but he insists upon it that it is not constant.

Doubts are entertained by some practitioners of the possibility of obtaining bony union in cases of transverse fracture of the olecranon. Mr. Earle thinks it would be obtained in every instance, provided the broken surfaces were steadily maintained in correct apposition. This correct apposition, he believes, might be obtained in the majority of recent cases, though it would be difficult to accomplish it in cases which had been neglected for some time, and where considerable retraction had taken place, accompanied by inflammation and swelling. In such cases, indeed, he questions whether ligamentous, is not preferable to bony union.

"It remains only to speak of the mode of treatment best adapted to fulfil the several indications. As far as my own experience goes, a slight degree of flexion of the elbow, to the extent of about 160 degrees, is the best position, and will admit of the nicest coaptation of the fractured surfaces; whilst, at the same time, it is the least irksome to the patient." 166.

After objecting to the plan of treatment laid down by Sir Astley Cooper and others, Mr. Earle gives his own, in the following words.

"After confining the lateral motion of the upper broken portion with a slight compress, and straps of adhesive plaster carried obliquely across the elbow, and having accurately adapted the broken surfaces together, I form a case of strong pasteboard, softened with hot water: this is applied in two pieces, about a foot long; one in front, and the other at the back of the arm, which are bound with a circular roller to the arm, bent to an angle of 160 degrees. This is left on until dry, during which time the patient remains in a recumbent posture, with the arm on a pillow. The pasteboard is then removed, and covered with wash-leather, which is glued over the surface; this gives it a great degree of solidity, and forms a very light and commodious case, sufficiently strong to resist any attempt at motion in the joint, and to protect it from any blows or injuries. With the assistance of this apparatus it is not necessary to confine the patient after a few days. With a view to prevent any swinging motion of the arm, and to afford additional security against accidental blows, I have found it advantageous to have a portion of ribbon attached to the front of the patient's dress, with a loop for the thumb or wrist to hang in when in exercise. After a fortnight, or three weeks, slight passive motion may be given to the joint, which will greatly accelerate the patient's ultimate recovery." 169.

The last original paper which we have to notice in this volume is on "Injuries near the Shoulder-joint." Mr. Earle's first observations are on injuries of the clavicle. In fractures occurring at the scapular extremity of this bone, little or no displacement can take place in the fractured parts, except from the external violence that produced the fracture; but, when the fracture is between the coracoid process and sternum, very considerable displacement may take place, partly from the action of the clavicular portion of the sterno-cleido-mastoideus muscle elevating the sternal portion of the clavicle, whilst the weight of the whole upper extremity depresses and carries forwards the scapular portion.

"In the treatment of these cases the principal indications are to elevate the shoulder even, in some cases, beyond its natural level, to allow for the action of the sterno-cleido-mastoideus muscle; to keep the shoulder drawn outwards from the body, by a wedge in the axilla; and to maintain the whole limb in a perfectly passive state, as every motion of the arm and scapula must be immediately communicated to the fractured part." 177.

The figure of 8 bandage is the common application in this country.

"The only effect of these bandages is to keep back the shoulders; but, at the same time, the scapula is pressed towards the sternum, and the fractured portion of the clavicle, being connected with it, is forced under the sternal portion. When a common linen bandage is employed, the tightness with which it is applied causes most distressing excoriations of the axilla; and generally some folds of it press so much on the scapular end of the clavicle as to depress it, and thus produce the very defect which it is intended to remedy. In the employment of every form of this bandage, the weight of the whole upper extremity remains unsupported, and it is necessary that a sling should be superadded to support the arm; which, as far as it goes, forms the most important part of the treatment; but common slings are very insecure, they always allow of considerable motion of the arm, and are quite at the patient's disposal to remove or not." 178.

Desault and other later French writers, employ between twenty and thirty yards of bandage, curiously and neatly applied, so as to secure the limb in position during cure, and so effectually to unite the whole extremity to the chest, that they move together and form, as it were, one body. But this is a very troublesome process—very irksome to the patient—and very easily deranged. Mr. Earle, therefore, devised, with his usual ingenuity, an apparatus which he has found very useful, not only in fractures of the clavicle, but in various other injuries occurring near the shoulder-joint.

"The apparatus consists of a strong sleeve, made of double jean, or linen cloth, which reaches from half-way up the upper arm, is fitted to the elbow, when bent to an angle of about 75 degrees, and terminates, like the sleeve of a straight waistcoat, in a cul-de-sac. This is applied to the arm, and secured by straps, or a lace and eyelet holes: at the extremity of this sleeve a band of strong webbing is attached, which is passed round the body, and fixed to a broad buckle, which is fastened to a belt of calf-skin, lined with wash-leather, about three inches broad, which is passed round the injured arm, just below the insertion of the deltoid. The action of this sleeve and strap is to prevent any motion in the arm or forearm, and to bind it firmly to the trûnk. To support the elbow in any position which may be required, I employ a leather cap, adapted to the extremity of the elbow, and hollowed out at its centre for the olecranon. This is put on over the sleeve, and from it two broad bands of webbing pass obliquely up to the opposite shoulder; one in front of the thorax, and the other behind. These bands are affixed to two broad buckles, which are attached to a leather shoulder cap, made of calf-skin, well padded and lined with wash-leather, which is adapted nicely to the shoulder by means of a buckle and strap, which passes under the axilla. By tightening or slackening these bands, the elbow may be either confined close to the side, or brought forward, as in the position required for fractures of the clavicle or the coracoid process; and it may be permanently and steadily fixed in that position. Another strap may be brought down from the anterior oblique strap, and passed round the wrist, to assist in supporting the weight of the extremity." 188.

Two papers more conclude the volume. These are republications—one "On the Re-establishment of a Canal in the Place of a Portion of the Urethra which had been destroyed"—the other, "On the Mechanism of the Spine." These have been already noticed in the pages of this Journal.

We have now presented to our readers a very full analysis of Mr. Earle's work, controversial, critical, practical, and mechanical. If we have, in a former number, had occasion to differ with our author on some controverted points of surgery, it was with great reluctance, for we hate controversies; and we can assure Mr. Earle, that we are always happy to meet him in the character of an ingenious and intelligent surgeon, advancing his science with zeal and ability.

## Figures and Tables

**Figure f1:**